# Supplementary material for: Splice-Junction-Based Mapping of Alternative Isoforms in the Human Proteome
Source: Cell Rep. Author manuscript; Available in PMC 2020 Jan 15. (PMC6961840; doi:10.1016/j.celrep.2019.11.026)
Supplement: 3 [file NIHMS1546469-supplement-3.zip › DF2/PXD006675/RightAtrium-196-Q8WZ42-SNCTVSVHVSEPATIVEKPESIK.pdf]

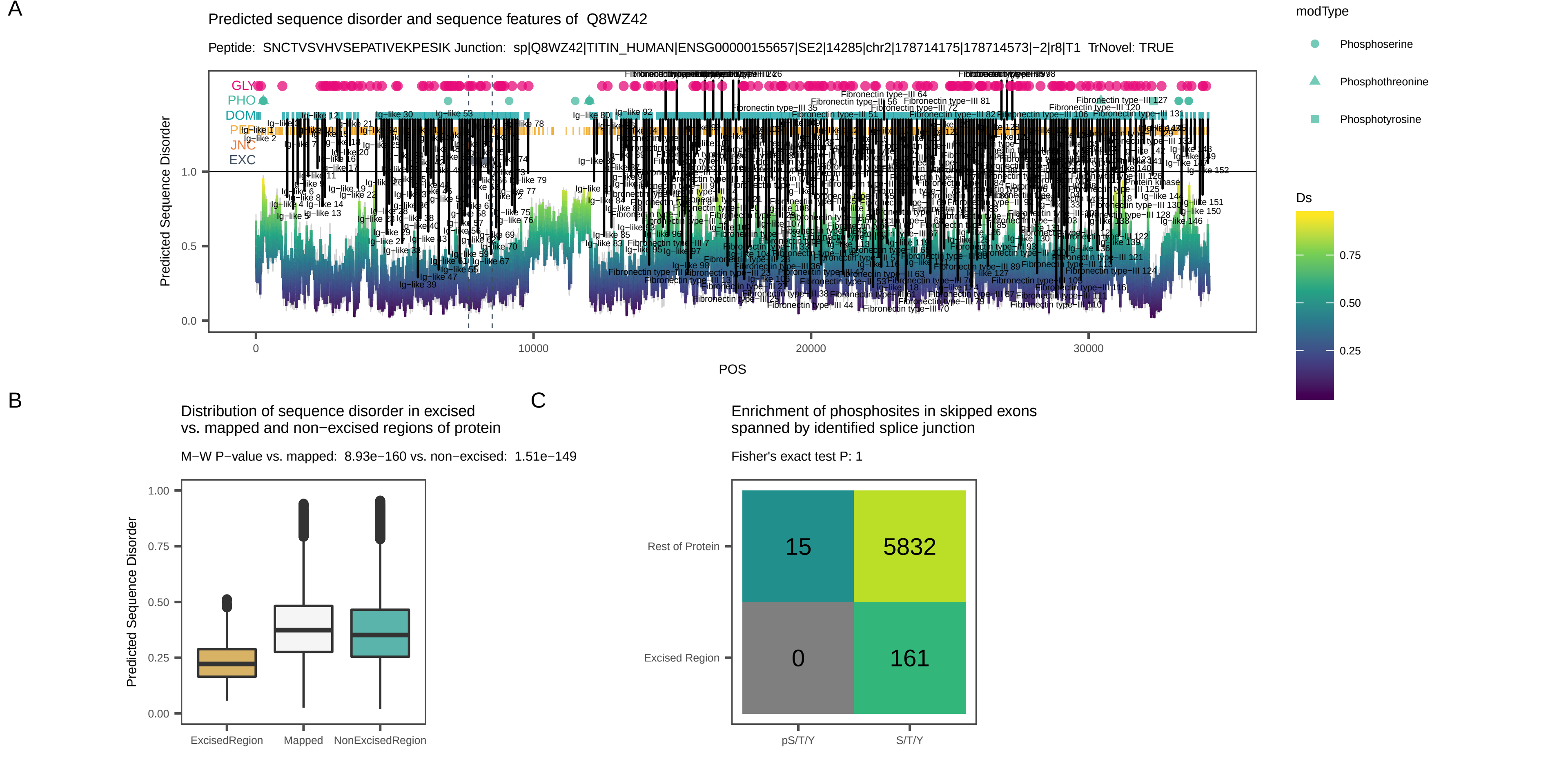
A
Predicted sequence disorder and sequence features of Q8WZ42
Peptide: SNCTVSVHVSEPATIVEKPESIK Junction: sp|Q8WZ42|TITIN_HUMAN|ENSG00000155657|SE2|14285|chr2|178714175|178714573|−2|r8|T1 TrNovel: TRUE
GLY
PHO
DOM
PEP
JNC
EXC
Predicted Sequence Disorder
1.0
0.5
0.0
0
10000
20000
30000
POS
modType
Phosphoserine
Phosphothreonine
Phosphotyrosine
Ds
0.75
0.50
0.25
B
Distribution of sequence disorder in excised vs. mapped and non−excised regions of protein
M−W P−value vs. mapped: 8.93e−160 vs. non−excised: 1.51e−149
Predicted Sequence Disorder
1.00
0.75
0.50
0.25
0.00
ExcisedRegion
Mapped
NonExcisedRegion
C
Enrichment of phosphosites in skipped exons spanned by identified splice junction
Fisher's exact test P: 1
Rest of Protein
Excised Region
15
5832
0
161
pS/T/Y
S/T/Y
